# The Metropolitan Hospital Sunday Fund

**Published:** 1903-12-19

**Authors:** 


The resolution was then put and carried unanimously.

### THE METROPOLITAN HOSPITAL SUNDAY FUND.

### ANOTHER RECORD YEAR.

On Tuesday, the 15th inst., the annual general meeting of the constituents of the Metropolitan Hospital Sunday Fund was held in the Mansion House, the Lord Mayor, Sir James Ritchie, in the chair. There was an exceptionally large attendance.

### The Report.

The Council, in their report for the year ended October 31st last, stated that the total amount collected was £64,975, the largest sum ever collected on Hospital Sunday since its foundation. Mr. George Herring had again generously added one-fourth to the amount collected in places of worship, and his contribution had been £12,302. The number of congregations which had contributed showed an increase of 62. An invitation to join in the Fund had been sent to most of the clergy in the home counties, but only 20 congregations responded. The Council thought that it could not be too often repeated that these country districts furnished a large proportion of the in-patients of our hospitals.

Sir Joseph Savory, in moving the adoption of the report, congratulated the Council upon another record year. He hoped that, although the sum collected was large, they would exceed it next Hospital Sunday.

The Chief Rabbi (Dr. Adler) seconded the motion. He said that at no time were the needs of the hospitals more urgent. Enhanced cost of upkeep was necessitated by the demands of medical science, and great expenditure was being incurred so as to bring both the buildings and appliances into line with present day needs. Appeals for close on a million pounds were to be made in the immediate future for reconstruction schemes, and they must not slacken their efforts for that Fund lest the silent hospitals which did not make special appeals should suffer.

A Proposed Amendment.

The Hon. Stephen Coleridge moved the following amendment:—

That it be an instruction to the Committee in future to deduct from the amount allocated to the general funds of any hospital such sums as may have been transferred from those general funds to the funds of any school or college during the preceding year.

The Lord Mayor said he was informed that that had always been done; that the money handed over to each hospital was less any sum paid to its medical school, the amount being deducted before the award was decided on.

Mr. Richard Biddulph Martin, one of the hon. secretaries of the Fund, said he could quite confirm what the Lord Mayor had said. It was only to the support of the philanthropic side of the hospitals that the Fund contributed.

Mr. Coleridge asked to be allowed to say a word in support of his amendment. What he wanted to urge was that the immense sum of money subscribed by the charitable public imposed on those who collected it the duty of criticising those who spent it. Four of their largest hospitals diverted from the money given them for the relief of the sick poor sufficient to support 70 or 80 beds now closed for lack of funds, and gave it to their medical schools. These schools were for the education of students whose fathers ought to pay for that education. He thought that cast on the Committee the duty of expressing disapproval of such diversion and that they should declare they would not support these hospitals so much as they would if it ceased.

The Ven. Basil Wilberforce, Archdeacon of Westminster, whose name had been handed in as seconder to the amendment, said that if as they were assured these amounts were deducted, the amendment fell to the ground, and he was perfectly satisfied. He certainly felt that the noble profession of medicine ought not to accept for the education of its alumni money subscribed for the relief of the sick-poor.

A further question was put as to whether they were to understand that if to a hospital a grant of £2,000 was made, and that same hospital paid £900 to its medical school, that the next year the grant would be reduced to £1,100? And to this the reply was given that it had always been the case that in proportion to what was paid to medical schools so were the grants reduced.

The report was then adopted unanimously.

The Rev. R L. Allwork then moved:—

That the Council for the year 1903 be re-elected for the year 1904, with the Rev. H. R. Gamble, the Rev. Archibald Fleming, the Rev. Frank Freeston, the Rev. J. Scott-Lidgett, Sir Marcus Samuel, and Mr. Arthur T. West to fill vacancies.

Mr. Dibdin seconded.

A constituent said he rose to support the resolution, but he wished to ask that his district, a very poor one, which had subscribed two guineas made up of farthings and halfpence, should have a few more letters than in strict accuracy it was entitled to. He also hoped that representations would be made to the hospitals in respect of the long time which he said poor people had sometimes to wait before receiving attention in the out-patient departments. The loss of time was very serious to them.

Sir Henry Burdett, K.C.B., said that with a hospital, as with a West End consultant, waiting was a question of popularity. As paying patients they all knew they might have to wait hours to see an eminent medical man, with this great disadvantage, as compared with a hospital patient, that after they had waited in the consulting room a couple of hours or more they might have a servant appear and tell them that the eminent doctor regretted that he could see no more patients that day. No poor man attending at a hospital was ever treated so. The hospitals regretted that the demand was so great, but everything was done to expedite matters, and so to sort the patients as to lessen the waiting as much as possible. In many hospitals refreshment counters had been set up in the out-patient departments, and this had greatly added to the comfort of the patients and sufficiently indicated the spirit in which the matter was being dealt with.

The resolution was then carried.

Mr. George Herring's Gift.

The Right Hon. Sir Savile Crossley, M.P., moved—

That the best thanks of this meeting be given to George Herring, Esq., for his generosity, which has assisted the Fund to secure the record amount of £64,975.

They all knew, he said, the good that had been done by Mr. George Herring, and that he had been one of the principal factors in making this a record year. London and its needs were growing, and he hoped the amount to be collected in the present year would show a still further increase. He was glad to know that the three great funds of which he was a member—the King's, the Saturday, and the Sunday Funds—were all working side by side in the greatest harmony.

The Earl of Meath seconded. He said Mr. Herring was wise in the form of his gift, for by adding a quarter to everything subscribed he stimulated the gifts of those who could give only small sums, but whose number was great. They must not forget that the hospitals were still greatly in need of money—need so great that many were saying that we should have to fall back on the rates or on Imperial taxes. Having lived abroad and seen the Continental hospitals he should be very sorry if that came to pass. There were strong reasons against it which it was difficult to explain here; but for the sake of the moral courage of the people he was desirous of depending on voluntary contributions.

The resolution was adopted with acclamation.

Hospital Sunday, June 12th, 1904.

The Venerable Archdeacon of London, (Dr. Sinclair) moved:—

That the 12th of June be fixed for Hospital Sunday of 1904, and that the cordial co-operation of all ministers of religion within the metropolitan area be again invited in the usual way.

He said the co-operation for which they asked had been most successful, in spite of most deplorable weather that year, chiefly, he thought, through the heroic efforts of their secretary, Sir Edmund Hay Currie. He it was who suggested the Royal visit to St. Paul's, which resulted in their raising twenty times as much as before. He was glad that the great Cathedral at last led the way. He noted with pleasure that there were 62 more congregations contributing, and hoped there would soon be no congregation which did not send in something. They had appealed this year to the country Churches, for it was a fact that some 25 per cent. of the patients in the London hospitals came from outside. Twenty congregations had responded, so he hoped their secretary would not despair. It was a great pleasure to meet there on common ground, despite their regret at so much that separated them for the time, and it could not but be of the greatest benefit to them all so to meet

The Rev. W. H. Harwood, of Union Congregational Chapel, Islington, reciprocated the Archdeacon's pleasure that they could meet there on the broad ground of humanity, and wished it were oftener that they did so.

A Fair Field for Hospital Week.

Sir Henry Burdett moved :—

That the laws of the Constitution, which have been in force during the past year, be continued with the following addition to Law IX. "And in the further event of a particular hospital, dispensary, or institution, fixing any day or days in the week immediately preceding Hospital Sunday in any year for the purpose of pressing the claims of such institution upon the public, it shall be an instruction to the Committee of Distribution, when determining the award to such institution, to take into consideration the amount received from such an appeal and the loss, if any, resulting therefrom to the Fund."

He said if there was one thing in connection with the Fund they all desired it was that London should on Hospital Sunday contribute £100,000 to the hospitals. One of the means adopted to this end had been to call in the assistance of the laity by seeking the assistance of the editors of the metropolitan newspapers and organising an effort during the six days preceding Hospital Sunday to arouse all London. They had gone on prospering because the three great Funds recognised the principle that there should be no overlapping, and they expected that no single institution should depart from that understanding by selecting Hospital Sunday week for the purpose of urging particular claims. Unfortunately they had found that three whole days in that week had been taken up on behalf of one or two hospitals which so might use the general interest raised by the newspaper appeals on behalf of all the hospitals for their own benefit. The Council therefore fell back on Law IX, which provided that—

In the event of a congregational collection, made on Hospital Sunday, being given to a particular hospital, dispensary, or institution instead of being sent to the General Fund, the amount so sent shall be deducted from the grant made to that hospital, etc, and can in no case be included as forming part of the Fund.

And all the addition he was now moving did was to make it plain that all hospitals were placed on an identical footing. He hoped it would be realised that it was not only just but necessary to enforce this. They wanted united action in the cause of all by all participating charities, and without it they would never be in a position to raise £100,000, which was the goal they set before them.

Sir William Church, K.C.B., M.D., said that after the clear explanation to which they had just listened it was needless for him to speak further. It had always been their intention and desire that all hospitals should share equally in proportion to their need, and it was exceedingly unfair for any hospital to try to reap any special benefit.

The constituent who had previously referred to the waiting in out-patient departments wished to express objection to the Sunday parades in the East End, but was ruled out of order, the subject not being relevant to the motion under discussion, which was then put and carried unanimously, and a hearty vote of thanks to the Lord Mayor, proposed by Prebendary Ridgeway and seconded by Mr. R. B. Martin, terminated the proceedings.

